# Activation of MEK2 is sufficient to induce skin papilloma formation in transgenic zebrafish

**DOI:** 10.1186/s12929-015-0207-2

**Published:** 2015-11-17

**Authors:** Chih-Ming Chou, Yi-Chung Chen, San Su, Gen-Der Chen, Kai-Yun Huang, Huang-Wei Lien, Chang-Jen Huang, Chia-Hsiung Cheng

**Affiliations:** Department of Biochemistry and Molecular Cell Biology, School of Medicine, College of Medicine, Taipei Medical University, Taipei, 110 Taiwan; Graduate Institute of Medical Sciences, College of Medicine, Taipei Medical University, Taipei, 110 Taiwan; Institute of Biological Chemistry, Academia Sinica, 128 Academia Rd., Sec 2, Taipei, 115 Taiwan; Department of Biochemistry, School of Medicine, College of Medicine, Taipei Medical University, 250, Wuxing Street, Taipei, 11013 Taiwan

**Keywords:** MEK2, Skin, Papilloma, Proliferation, Zebrafish

## Abstract

**Background:**

Mutations in mitogen-activated protein kinase (MAPK) kinase 1 (MEK1) that occur during cell proliferation and tumor formation are well described. Information on the roles of MEK2 in these effects is still limited. We established a constitutive MEK2 transgenic zebrafish, Tg(krt14:MEK2S219D-GFP), to elucidate the role of MEK2 in skin tumor formation.

**Results:**

We found that both constitutive MEK2 and MEK1 are able to phosphorylate the extracellular signal-regulated kinase 1 (ERK1) protein. Transient expression of constitutive MEK2 and MEK1 in the zebrafish epidermis induced papillary formation at 48 h post-fertilization, but no effects were observed due to the expression of MEK1, MEK2, or the dominant negative form of MEK2. The transgenic zebrafish, Tg(krt14:MEK2S219D-GFP), developed skin papillomas in the epidermis within 6 days post-fertilization (dpf). The phospho-ERK signal was detected in section of skin papillomas in an immunohistochemical experiment. Treatment with 50 μM of the MEK inhibitor, U0126, had significantly decreased the skin papilloma formation in Tg(krt14:MEK2S219D-GFP) zebrafish by 6 dpf. *In vitro* and *in vivo* proliferation assay in COS-1 cells and in Tg(krt14:MEK2S219D-GFP) transgenic fish show significantly increased cell number and Ki-67 signaling.

**Conclusion:**

Our data indicate that MEK2 is sufficient to induce epidermal papilloma formation through MAPK signaling in zebrafish, and this transgenic model can be used as a new platform for drug screening.

## Background

The Raf/mitogen-activated protein kinase (MAPK) kinase (MEK)/extracellular signal-regulated kinase (ERK) cascade is the downstream pathway of Ras which transforms its effects on its targets [15]. When Ras is activated by a mutation, it was hypothesized that downstream components will be constitutively activated and corresponding abnormal gene expressions will occur that drive proliferation. Several model systems of skin tumors in mice have been established [1–5, 7, 8, 12, 20]. Zebrafish are a very useful and convenient model organism for studying human diseases. So far, no squamous skin cell tumor model has been established in zebrafish [9, 10, 16, 25, 29].

MEK2 was reported to be a factor related to invasion and metastasis of pancreatic tumors [28]. The expression of Raf22w, v-Ha-Ras, or v-Src resulted in the constitutive activation of MEK1 and MEK2 [11]. A mutation of p21ras in hepatocellular carcinoma (HCC) was associated with marked changes in expression of the function of MAPK cascades mainly through MEK1 [19]. MEK1 is important for skin tumor development, and MEK2 could not compensate for this process with the loss of MEK1 function in a mouse model [27]. The MEK1 and MEK2 signaling pathways were not redundant or interchangeable for cell proliferation in SK-MEL-28 melanoma cells [17].

However, it is unclear if MEK2 constitutively activated in zebrafish is sufficient to induce epidermal neoplasia. Herein, we established a stable transgenic zebrafish model with consistent expression of MEK2^S219D^ under an epidermal-specific Krt14 promoter using the Tol2-transposone system to investigate the effects of MEK2^S219D^ on inducing epidermal neoplasias *in vivo*. Further treatment with U0126 in this transgenic zebrafish tumor model was used to evaluate the cause of skin papilloma. We suggest that this model can serve as a useful platform for cancer drug screening.

## Methods

### Fish

Zebrafish (*Danio rerio*) were maintained at 28 °C on a 14-h light/10-h dark cycle. Embryos were incubated at 28 °C, and different developmental stages were determined according to the description in the *Zebrafish Book* [30].

### Total RNA isolation

Total RNA was isolated from fertilized eggs (a pool of embryos at 12, 24, 36, 48, 72, 96, 120, and 144 h post-fertilization; hpf), using the RNAzol reagent (Tel-Test, Friendswood, TX, USA) according to instructions of the manufacturer.

### Isolation of the full-length *mek1*, *mek2*, and *erk1* cDNAs of zebrafish

cDNAs encoding the complete open reading frame (ORF) of zebrafish *mek1/2* and *erk1* were obtained by polymerase chain reaction (PCR) amplification using gene-specific primers according to the NCBI’s zebrafish EST database. PCR amplification was performed in a 50-μl reaction mixture containing 2 μl of first-strand cDNA, 0.5 μg of a forward primer (MEK1-F: 5′-CGG GAT CCA TGC AGA AAA GGA GGA AG-3′; MEK2-F: 5′-TAT TGG ATG TCA GTA GAG ACA ACC TGG-3′; and ERK1-F: 5′-CGG ACA GAA ACG ATG GCG GAA TCG-3′) and reverse primer (MEK1-R: 5′-GGG AAT TCC ATT CCC ACA CTG TGA GT-3′; MEK2-R: 5′-TTC AGC GCT ATG AGT GGG TGT GCT AGG-3′; and ERK1-R: 5′-CGT GAT GAC TGT CCC TCT CAG GAG-3′), 1.5 mM MgCl_2_, 0.2 mM dNTP, and 2.5 units Ex*Taq* (Takara Shuzo, Shiga, Japan). Samples were incubated in a thermal cycler (SensoQuest, Göttingen, Germany). PCR amplification was performed; the DNA fragment of MEK1 and MEK2 was cloned into a pGEM-T Easy vector (Promega, Madison, WI, USA) as pGEMT-MEK1 and pGMET-MEK2 plasmids, and the resultant clones were subjected to a sequencing analysis.

### Site-directed mutagenesis of zebrafish MEK1 and MEK2 cDNA

Site-directed mutagenesis was performed to generate plasmids encoding MEK1/2 mutants (S219D and S223A) using the pGEMT-MEK1 and pGEMT-MEK2 plasmid as a template for PCR amplification using specific oligonucleotide primers. The corresponding oligonucleotides used for PCR reaction were as follows with the altered bases underlined: MEK1S219D-F: 5′-GGA CAA CTC ATT GAC GAC ATG GCC-3′, MEK1S219D-R: 5′-GGC CAT GTC GTC AAT GAG TTG TCC-3′; MEK2S219D-F: 5′-CAG CTC ATC GAC GAT ATG GCC AAC TCC-3′, MEK2S219D-R: 5′-GGA GTT GGC CAT ATC GTC GAT GAG CTG-3′; and MEK2S223A-F: 5′-ATG GCC AAC GCC TTC GTT GGA ACA CGG-3′, MEK2S223A-R: 5′-CCG TGT TCC AAC GAA GGC GTT GGC CAT-3′. Sequences of the resultant plasmids were verified using DNA sequencing.

### Construction of the expression plasmids

The 4-kb upstream region of the zebrafish krt14 gene (krt14 promoter) was amplified from a BAC clone, DKEYP-113D7 (Source Biosicence, Nottingham, UK), and inserted into the pGEM-T Easy cloning vector (Promega, Madison, WI, USA) as pGMET-krt14-pro plasmid. The krt14 promoter was then inserted into the pTol2-GFP vector [14] at appropriate sites to generate the pTol2-krt14-GFP plasmid. MEK1, MEK1S219D, MEK2, MEK2S219D, MEK2S223A, and ERK1 were inserted into pcDNA3-mCherry, pcDNA3-GFP, and pcDNA3-HA from PCR products as pcDNA3-MEK1-mCherry, pcDNA3-MEK1S219D-GFP, pcDNA3-MEK2-GFP, pcDNA3-MEK2S219D-GFP, pcDNA3-MEK2S223A-GFP, pcDNA3-ERK1-HA and then inserted into the pTol2-krt14-GFP vector to generate pTol2-krt14-MEK1-mCherry, pTol2-krt14-MEK1S219D-GFP, pTol2-krt14-MEK2-GFP, pTol2-MEK2S223A-GFP, and pTol2-krt14-MEK2S219D-GFP plasmids. The pcDNA3-GFP and pcDNA3-mCherry plasmids were constructed by inserting cDNA corresponding to the green fluorescence protein (GFP) and mCherry coding-region into pCMV-HA-YUN, which was a gift from Dr. H.J. Kung (Davis Cancer Center, University of California, Sacramento, CA, USA).

### Microinjection of expression plasmid into zebrafish embryo

The expression plasmid was adjusted to a final concentration of 100 μg/ml in 1× Danieau solution (5 mM HEPES at pH 7.6, 58 mM NaCl, 0.7 mM KCl, 0.4 mM MgSO_4_, and 0.6 mM Ca(NO_3_)_2_) containing 0.5 % phenol red and injected into a zebrafish embryo in the one-cell stage using the Narishige IM 300 microinjector system (Narishigi Scientific Instrument, Tokyo, Japan).

### Cell culture and plasmid transfection

Monkey kidney fibroblast COS-1 cells (ATCC CRL-1650; VA, USA) were cultured in high-glucose Dulbecco’s modified Eagle’s medium (DMEM; Hyclone, UT, USA), supplemented with 10 % fetal bovine serum (FBS; Hyclone, UT, USA) in a humidified atmosphere of 5 % CO_2_ at 37 °C. COS-1 cells were transfected with pcDNA3, pcDNA3-MEK1-mCherry, pcDNA3-MEK1S219D-GFP, pcDNA3-MEK2-GFP, pcDNA3-MEK2S219D-GFP, pcDNA3-ERK1-HA plasmids, using Lipofectamine Plus reagent (Life technologies, Grand Island, NY, USA) according to manufacturer’s manner. Culture cells were harvested at 48 h post-transfection and lysed with lysis buffer. The protein level was analyzed by western blotting.

### Immunoblot analysis

Total cell lysates from transfected cells were harvested and separated on 10 % sodium dodecylsulfate (SDS)-polyacrylamide gels and transferred to polyvinylidene difluoride (PVDF) membranes. Membranes were blocked with 5 % skim milk in phosphate-buffered saline (PBS) for 1 h at room temperature and then incubated at 4 °C with an anti-HA monoclonal antibody, anti-Actin polyclonal antibody (Santa Cruz, Dallas, TX, USA), and anti-mCherry polyclonal antibody (GeneTex, Hsinchu, Taiwan). Signals were detected using Immobilon Western Chemiluminescent HRP Substrate (Millipore, Billerica, MA, USA).

### Subcellular localization and image analysis

COS-1 cells transfected with pcDNA3-ERK1-HA alone or with pcDNA3-MEK1-mCherry, pcDNA3-MEK1S219D-GFP, pcDNA3-MEK2-GFP, pcDNA3-MEK2S219D-GFP, and pcDNA3-MEK2S223A-GFP for 48 h were fixed with 4 % paraformaldehyde and permeabilized in PBS with 0.1 % Triton X-100. Immunostaining was performed using an anti-HA antibody at 4 °C overnight, followed by incubation with a Cy2-conjugated goat anti-mouse antibody or Cy3-conjugated goat anti-mouse antibody for 30 min and DAPI (1 μg/ml) for 5 min at room temperature. Photoimages were prepared using an Olympus IX70-FLA inverted fluorescence microscope (Olympus, Tokyo, Japan) equipped with the SPOT system (Diagnostic Instruments, Sterling Heights, MI, USA).

### Histology and Immunohistochemistry

Larvae in which a skin papilloma had formed from Tg(krt14-MEK2S219D-GFP) at 6 days post-fertilization (dpf) were fixed with 4 % paraformaldehyde for 1 day and dehydrated with 100 % methanol. Specimens were embedded in paraffin, sliced and stained with hematoxylin and eosin (H&E) or incubated with 3 % serum at room temperature for 1 h before incubation with an anti-pERK polyclonal antibody (Santa Cruz, Dallas, TX, USA) or anti-Ki-67 polyclonal antibody (GeneTex, Hsinchu, Taiwan) containing 5 % serum and 2 mg/ml bovine serum albumin (BSA) at 4 °C overnight. After washing with PBST, embryos were incubated with a horseradish peroxidase (HRP)-conjugated goat anti-rabbit secondary antibody at room temperature for 3 h. HRP labeling was visualized using DAB (Merck, Darmstadt, Germany) as a substrate. Nuclei were counterstained with hematoxylin (Merck, Darmstadt, Germany).

### Chemical treatment and statistical analysis

Tg(krt14-GFP) and Tg(krt14-MEK2S219D-GFP) embryos were dechorionated at 1 dpf. Ten embryos with high GFP fluorescent signaling were transferred to each well in 1 ml water containing 50 μM U0126 (Sigma, MO, USA) or 0.01 % DMSO (Sigma, MO, USA) and incubated from 1 to 6 days at 28 °C incubator. The number of zebrafish forms any papilloma was assessed at 6 dpf under an Olympus IX70-FLA inverted fluorescence microscope. Results from three independent experiments are expressed as the mean ± standard deviation (SD). Statistical significance between groups was determined by an unpaired Student’s t-test. A probability of *p* < 0.001 was indicated as ***.

### Cell proliferation assay

Cell proliferation was performed by trypan blue assay. Briefly, 3x10^4^ COS-1 cells were seeded in 24-well plate overnight. COS-1 cells were transfected with 1 μg pcDNA3-GPF or pcDNA3-MEK2S219D-GFP plasmids using Lipofectamine Plus reagent (Life technologies, Grand Island, NY, USA) according to manufacturer’s manner. Culture cells were trypsinized and the number of viable cells was virtually counted with a hemocytometer at day 1, 2, 3 and 4 post-transfection. Three counts per well were performed on three different experiments. Statistical significance between groups was determined by an unpaired Student’s t-test. A probability of *p* < 0.05 was indicated as *.

## Results

### Cloning and characterization of the zebrafish *mek1* and *mek2 (mek2a)* cDNAs

**Fig. 1:**
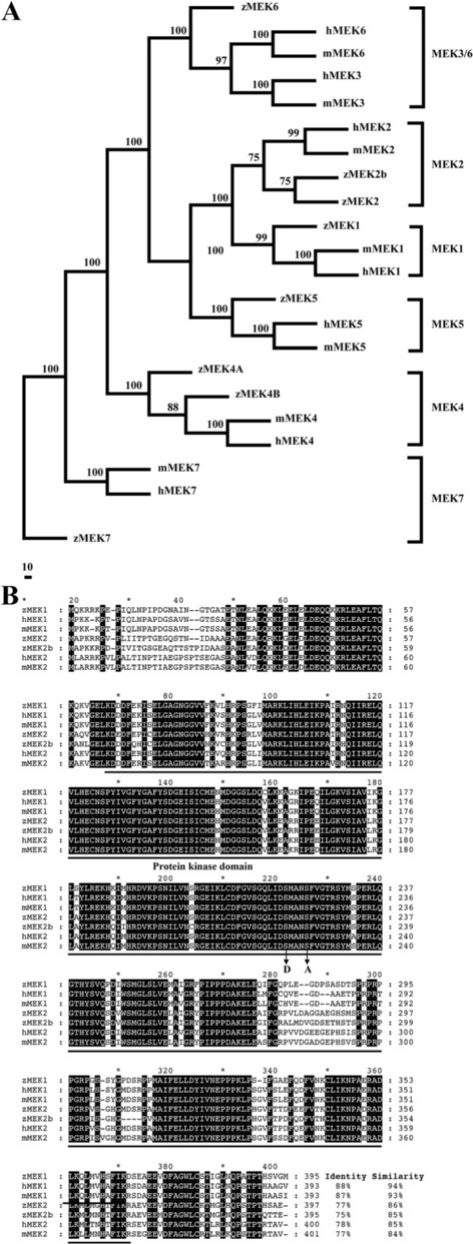
**a**. Phylogenetic analysis of the MEK gene family in the human, mouse, and zebrafish. Each member of the MEK family was separated into individual monophyletic groups. Amino acid sequences were aligned with ClustalW. Accession numbers for these sequences are as follows: zMEK1 (NP_998584), hMEK1 (NP_002746), mMEK1 (NP_032953), zMEK2 (zMEK2a, NP_001032468), zMEK2b (NP_001121753), hMEK2 (NP_109587), mMEK2 (NP_075627), hMEK3 (NP_659731), mMEK3 (NP_032954), zMEK4A (NP_991299), zMEK4B (NP_001082890), hMEK4 (AAH60764), mMEK4 (NP_033183), zMEK5 (NP_001107789), hMEK5 (AAA96146), mMEK5 (BAA82040), zMEK6 (NP_571799), hMEK6 (AAB05035), mMEK6 (AAH75652), zMEK7 (XP_689405), hMEK7 (O14733), mMEK7 (Q8CE90). **b**. Protein sequence alignment of zebrafish MEK1 and MEK2 with those of the human and mouse. The predicted protein kinase domain is indicated. The amino acid sequence of zebrafish MEK1 showed high conservation with human (88 % identity and 94 % similarity) and mouse MEK1. Zebrafish MEK1 shared 76 % identity and 85 % similarity in the protein sequence with MEK2. Mutation sites of the constitutively activated (S219D) and dominant negative (S223A) forms of zebrafish MEK1 and MEK2 are indicated. Accession numbers for these sequences are as follows: zMEK1 (NP_998584), hMEK1 (NP_002746), mMEK1 (NP_032953), zMEK2 (zMEK2a, NP_001032468), zMEK2b (NP_001121753), hMEK2 (NP_109587), mMEK2 (NP_075627)

The MEK family has 7 members, MEK1 ~ MEK7, in mammals. According to genomic predictions from NCBI zebrafish EST/cDNA, we identified 7 *mek* genes (*mek1*, *mek2*, *mek3*, *mek4a*, *mek4b*, *mek5*, and *mek7*) in zebrafish. Phylogenetic analysis of these 7 MEK proteins with mammalian counterparts indicated that only MEK3 but not MEK6 was found in zebrafish; instead, there is a duplication of MEK4 to form MEK4A and MEK4B. Each member of the MEK family was separated into individual monophyletic groups (Fig. 1a).

The *mek1* and *mek2* cDNAs were isolated from mixed cDNA pools prepared from different developmental stages of zebrafish embryos by PCR amplification. Full-length cDNA of zebrafish *mek1* and *mek2* were 1191 and 1194 base-pair long, encoding two proteins of 396 and 397 amino acid residues, respectively. The overall amino acid sequence of zebrafish MEK1 showed 76 % identity and 85 % similarity to that of MEK2. The zebrafish MEK1 respectively showed 88 and 87 % identities to human and mouse MEK1 and shown 78 % identities to human and mouse MEK2 at the level of amino acid sequences (Fig. 1b). The encoded amino acid sequence of zebrafish MEK1 and MEK2 was highly homologous to human and mouse proteins. The phosphorylated site of MEK1 was also conserved (Fig. 1b, arrow). These data suggest that zebrafish MEK1 and MEK2 are highly conserved with mammalian MEK1 and MEK2.

### Both MEK1 and MEK2 phosphorylated the ERK1 protein

**Fig. 2:**
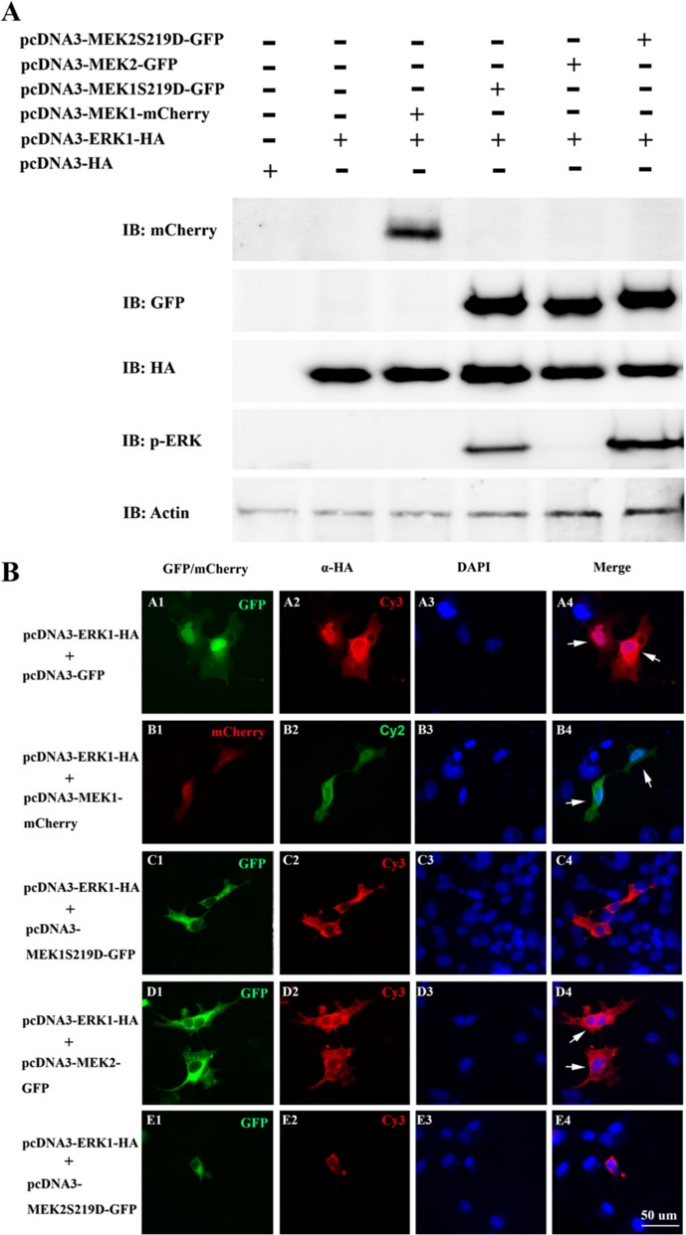
Constitutively activated MEK1 (MEK1^S219D^) and MEK2 (MEK2^S219D^) both phosphorylated ERK1. **a**. COS-1 cells were transiently transfected with pcDNA3-HA, pcDNA3-ERK1-HA, pcDNA3-MEK1-mCherry, pcDNA3-MEK1S219D-GFP, pcDNA3-MEK2-GFP, or pcDNA3-MEK2S219D-GFP. Total lysates were analyzed by Western blotting using anti-HA, anti-GFP, anti-pERK monoclonal antibodies; anti-mCherry and anti-Actin polyclonal antibodies. **b**. Intracellular localization of ERK1, MEK1, and MEK2 in COS-1 cells by fluorescent microscopy. The pcDNA3-ERK1-HA was co-transfected with pcDNA3-GFP (A1-A4), pcDNA3-MEK1-mCherry (B1-B4), pcDNA3-MEK1S219D-GFP (C1-C4), pcDNA3-MEK2-GFP (D1-D4), or pcDNA3-MEK2S219D-GFP (E1-E4). Cy2 or Cy3 dye used an anti-HA monoclonal antibody to detect localization of ERK1 as visualized. DAPI was used to stain nuclear DNA. White arrows indicate the ERK1 protein localized in nuclei and the cytoplasm. IB, immunoblot

In the MAPK signaling pathway, ERK proteins are phosphorylated by activated MEK1 and MEK2 to induce downstream gene transcription. Phosphorylation at serine 218 and 222 at human MEK1 by the Raf protein activated ERK1/2 phosphorylation. To elucidate the phosphorylation activity of zebrafish MEK1 and MEK2, we identified and cloned the *mek*1, *mek*2, and *erk*1 cDNAs from zebrafish embryonic cDNA. The human MEK1 phosphorylation sites of serine 218 and 222 were identified as serine 219 and 223 of zebrafish MEK1 and MEK2 (Fig. 1b). Site-directed mutations of S219D and S223A were performed at zebrafish MEK1 and MEK2 as MEK1^S219D^, MEK2^S219D^, and MEK2^S223A^. Expression plasmid constructs of zebrafish MEK1, MEK1^S219D^, MEK2, MEK2^S219D^, MEK2^S223A^, and ERK1 were constructed into the pcDNA3-mCherry, pcDNA3-GFP, and pcDNA3-HA plasmids to produce pcDNA3-MEK1-mCherry, pcDNA3-MEK1S219D-GFP, pcDNA3-MEK2-GFP, pcDNA3-MEK2S219D-GFP, pcDNA3-MEK2 S223A, and pcDNA3-ERK1-HA, respectively. Transfection of the pcDNA3-ERK1-HA plasmid individually or with the pcDNA3-MEK1-mCherry, pcDNA3-MEK1S219D-GFP, pcDNA3-MEK2-GFP, or pcDNA3-MEK2S219D-GFP plasmid into COS-1 cells was used to detect phosphorylation of ERK1. A Western blot analysis showed that MEK1 and MEK2 were unable to phosphorylate ERK1. In contrast, constitutively activated MEK1^S219D^ and MEK2^S219D^ could phosphorylate ERK1 (Fig. 2a). These data suggest that zebrafish MEK1 and MEK2 both exhibit ability to phosphorylate ERK1.

It has been shown recently that overexpression of the ERK protein without active signaling caused its accumulation in the nuclei and the cytoplasm. In the presence of the active MEK protein, the ERK protein was translocate from nuclei into the cytoplasm [18]. To further characterize the functions of MEK1 and MEK2, we examined the cellular localization of ERK1. In GFP, MEK1 or MEK2 expressed COS-1 cells, the ERK1 protein was accumulated in the nuclei and cytoplasm (Fig. 2b. A1-A4, B1-B4, D1-D4; arrow). In the presence of active MEK1^S219D^-GFP and MEK2^S219D^-GFP, the ERK1 protein was located in the cytoplasm (Fig. 2b, C1-C4, E1-E4). These results indicate that active zebrafish MEK1 and MEK2 both are able to induce ERK1 protein translocate from nuclei to the cytoplasm.

### Transient expression of MEK1^S219D^ and MEK2^S219D^ induced papillary formation in the zebrafish epidermis

**Fig. 3:**
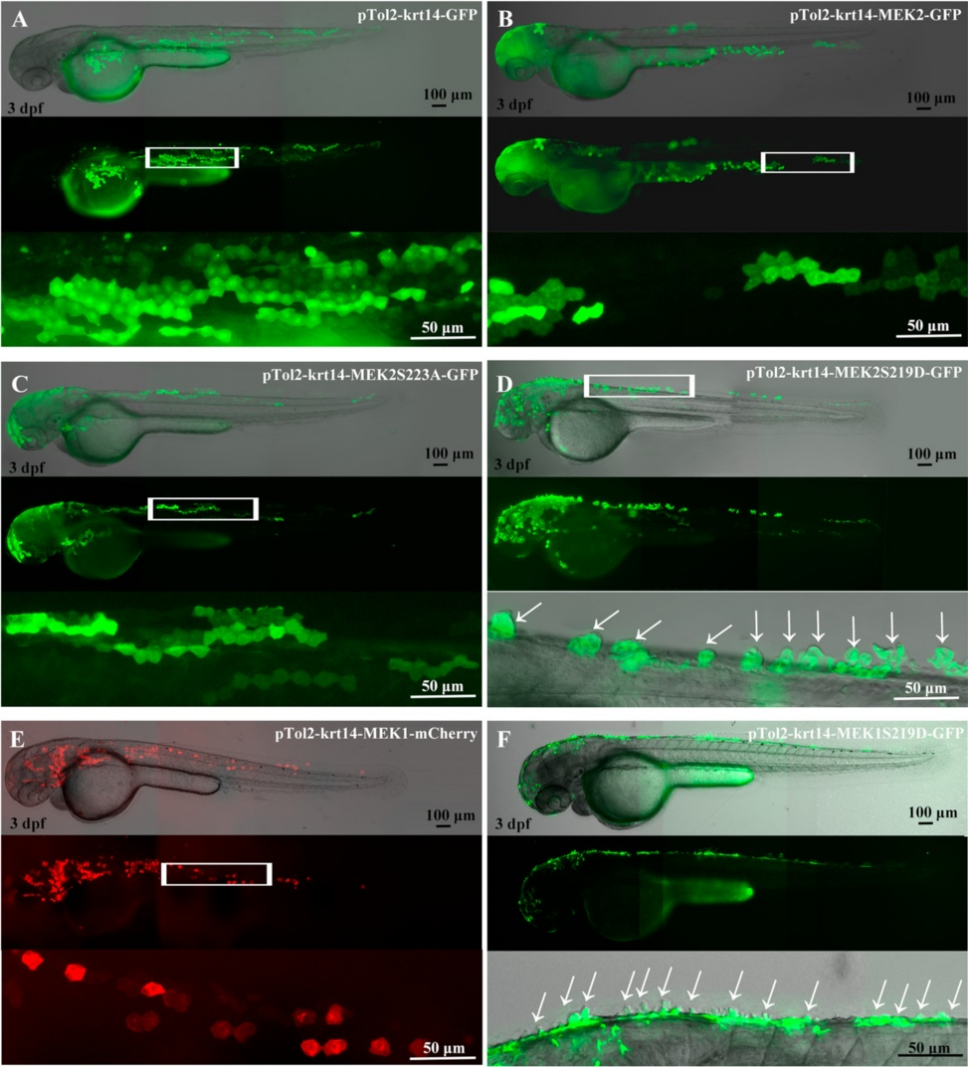
Transient expressions of MEK1 and MEK2 driven by the krt14 promoter induced papillae formation in skin cells. Lateral view of pTol2-krt14-GFP (**a**), pTol2-krt14-MEK2-GFP (**b**), pTol2-MEK2S223A-GFP (**c**), pTol2-krt14-MEK2S219D-GFP (**d**), pTol2-krt14-MEK1-mCherry (**e**), and pTol2-krt14-MEK1S219D-GFP (**f**) plasmids, which were microinjected into 1-cell stage of zebrafish embryos and visualized at 3 days post-fertilization (dpf). The arrow indicates skin cell papillae and budding in the upper epidermis. The upper panel is a fluorescent image. The middle panel is merged fluorescent and bright-field images. The lower panel is an enlargement of the white box in the middle panel

It was reported that constitutive activation of MEK1 could transform normal intestinal epithelial cells into tumors [7]. MEK1 and MEK2 were reported to be functionally redundant in epidermal development in mouse models [26]. MEK1 is important in skin tumor development, and MEK2 cannot compensate for loss of MEK1 function [27]. Another report indicated that MEK2 is sufficient for melanoma cell proliferation, but MEK1 is not [17]. To study the role of zebrafish MEK2 in the formation of skin tumors and comparison of expression with MEK1, pTol2-krt14-MEK2-GFP, pTol2-MEK2S219D-GFP, pTol2-MEK2S223A-GFP, pTol2-MEK1-mCherry and pTol2-MEK1S219D-GFP were microinjected into one-cell stage of zebrafish embryos and revealed the expression at 3 dpf. The zebrafish embryos injected with pTol2-krt14-MEK2-GFP (Fig. 3b), pTol2-Krt14-MEK2S223A-GFP (Fig. 3c), and pTol2-krt14-MEK1-mCherry (Fig. 3e) displayed specific GFP or mCherry expression in the skin cells (Fig. 3a). The pTol2-krt14-MEK2S219D-GFP-injected embryos also displayed specific expression in the skin cells. Furthermore, several skin cells in the upper epidermis were shown to have papillae and to have budded (Fig. 3d, arrows). The pTol2-krt14-MEK1S219D-GFP transgenic embryos also displayed papillae and budded skin cell formation in the epidermis (Fig. 3f). These results suggest that active zebrafish MEK2 and MEK1could promote the proliferation of skin cells to form papillae.

### MEK2 is sufficient to induce skin papilloma formation in transgenic zebrafish

**Fig. 4:**
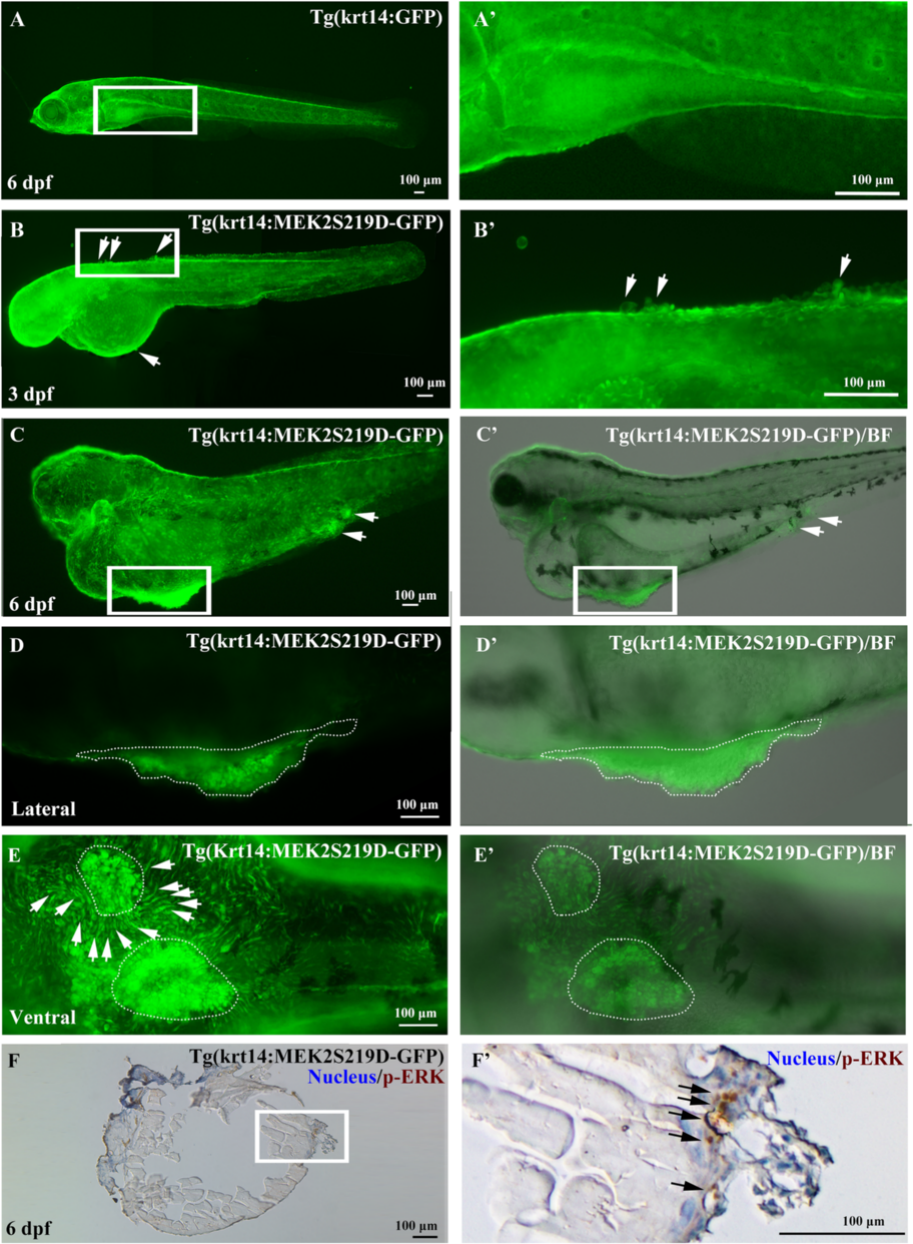
Skin tumor formation in Tg(krt14: MEK2S219D-GFP) zebrafish. Skin cells proliferated and formed papilla from 3dfp (**b**, *B*’, white arrow) on the yolk skin in Tg(krt14: MEK2S219D-GFP) embryos to form a skin tumor at 6 dpf (**c**, and *C*’; lateral view, **d** and *D*’, white-dots area; ventral view, **e** and *E*’, white-dots area). Tg(krt14:GFP) embryos had developed normal skin cells at 6 dpf (**a**, *A*’). An immunohistochemical experiment was used to detect ERK phosphorylation in skin tumors. p-ERK was detected by a p-ERK monoclonal antibody and visualized by DAB (brown). Nuclei were counterstained with hematoxylin (blue) (**f**, *F*’)

To further elucidate the tumorigenesis of MEK2 in zebrafish, we established a transgenic zebrafish known as Tg(krt14:MEK2S219D-GFP) in which expression of the MEK2S219D-GFP fusion protein was driven by the krt14 promoter to establish a skin tumor animal model. The skin papilloma formation of Tg(krt14:MEK2S219D-GFP) was presented approximately 34 % in homozygous and 5 % in heterozygous. Tg(krt14:MEK2S219D-GFP) zebrafish expressed the GFP signal from embryos to adult. Tg(krt14:MEK2S219D-GFP) zebrafish exhibited skin papilla from 3 dpf (Fig. 4b-B’) and then developed to a skin tumor at 6 dpf (Fig. 4c-C’, d-D’, e-E’; white-dots area). In addition, we established a transgenic line known as Tg(krt14:GFP), in which GFP expression was driven by the krt14 promoter to reveal the expression pattern of skin cells. Homozygous Tg(krt14:GFP) transgenic zebrafish exhibited the GFP signal in the entire skin cells, which was a normal phenomenon from embryos to adult (Fig. 4a-A’). These results indicated that constitutive MEK2 was sufficient to induce skin papilloma formation.

To further examine whether skin papilloma formation induced by MEK2 is through downstream signaling activation, we tested ERK phosphorylation of skin papilloma by an IHC experiment. Phosphorylated ERK is well known as a proliferation marker during tumor formation. Phosphorylation of ERK in skin papilloma of Homozygous Tg(krt14:MEK2S219D-GFP) larvae was detected by IHC experiment (Fig. 4f-F’). These results suggested that zebrafish constitutive activated MEK2^S219D^ induced skin papilloma formation through phosphorylation of downstream ERK signaling.

### U0126 inhibits skin papilloma formation induced by MEK2S219D

**Fig. 5:**
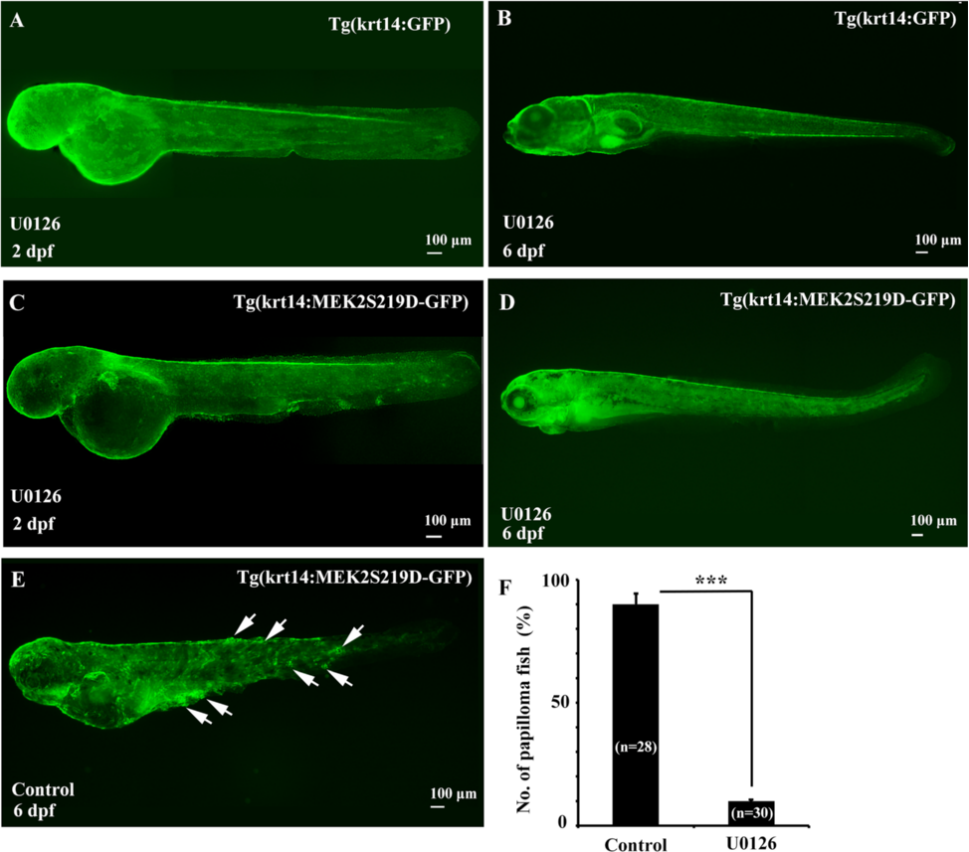
The MEK inhibitor, U0126, inhibited skin tumor formation in Tg(krt14: MEK2S219D-GFP) embryos. Tg(Ka14:GFP) embryos were observed by treated with 50 μM U0126 at 2 dpf (**a**) and 6 dpf (**b**). Tg(krt14: MEK2S219D-GFP) embryos were observed by treated with 50 μM U0126 at 2 dpf (**c**) and 6 dpf (**d**) or treated with DMSO at 6 dpf (**e**). The white arrow indicated skin papilloma. **f** The frequency of tumor formation in Tg(krt14: MEK2S219D-GFP) embryos incubated with DMSO (Control) or U0126

We further examined whether skin papilloma formation in Tg(krt14:MEK2S219D-GFP) zebrafish was induced by MEK2 activation by specific MEK1/2 inhibitor, U0126. Tg(krt14:MEK2S219D-GFP) transgenic zebrafish were incubated in 50 μM U0126 or 0.01 % DMSO (Control) from 1 to 6 dpf. No significantly abnormal phenotype of Tg(Krt14:GFP) zebrafish larvae was observed by treated with U0126 at 2 and 6 dpf (Fig. 5a, b). No skin papilloma was observed at 2 dpf in Tg(krt14:MEK2S219D-GFP) zebrafish larvae (Fig. 5c). Incubated Tg(krt14:MEK2S219D-GFP) zebrafish larvae with 50 μM at 2 dpf or 0.01 % DMSO as control, no skin papilloma was observed in U0126 treatment at 6 dpf (Fig. 5d) but several skin papilloma was observed in DMSO treatment at 6 dpf (Fig. 5e). The skin papilloma formation in transgenic zebrafish larvae was presented 90 % in DMSO treatment and 10 % in U0126 treatment at 6 dpf (Fig. 5f). These results suggest that the skin papilloma formation in Tg(krt14: MEK2S219D-GFP) zebrafish larvae can be inhibited by the specific MEK inhibitor, U0126. The MEK2S219D is sufficient to induce skin papilloma formation through activation of MAPK pathway.

### *In vitro* and *in vivo* cell proliferation assay in COS-1 cells and Tg(krt14:MEK2S219D-GFP) transgenic zebrafish

**Fig. 6:**
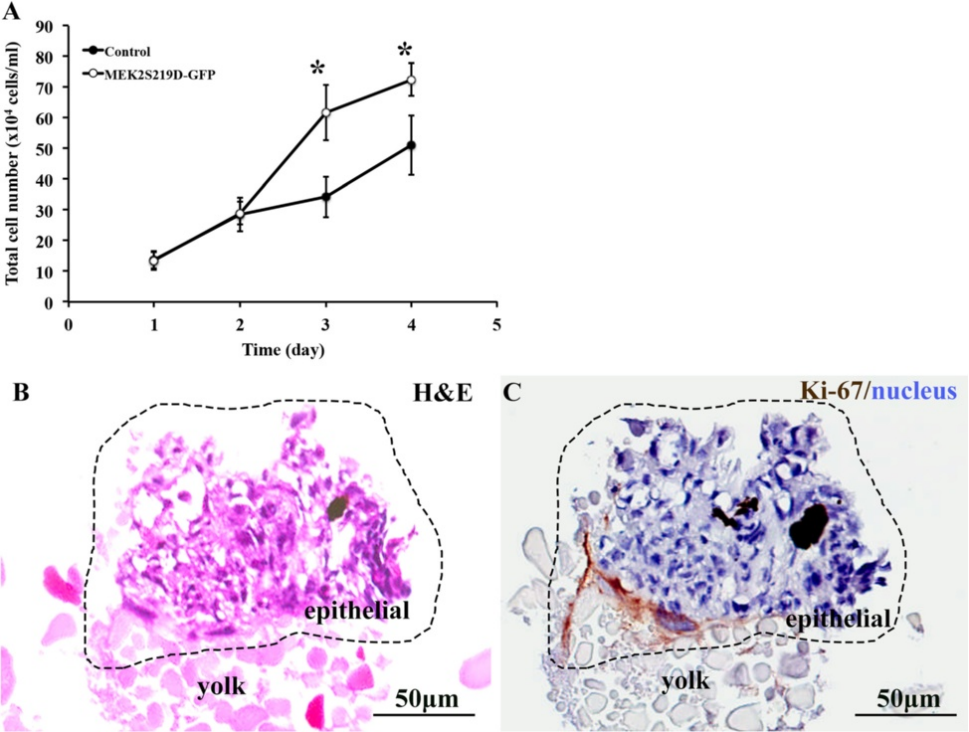
Effect of MEK2S219D on cell proliferation was analyzed in COS-1 cells and in transgenic zebrafish. **a** The pcDNA3-MEK2S219D-GFP and pcDNA3-GFP (control) plasmids was transfected to COS-1 cells and evaluated the cell numbers by trypan blue assay from 1 to 4 days post-transfection. Results represent the mean of three independent determinations. * *p* < 0.05. **b** H&E-stained section in papilloma (black-dots area) of transgenic zebrafish. **c** Ki-67 IHC staining in papilloma (black-dots area) of transgenic zebrafish

To further assess the effect of proliferation induced by MEK2S219D, we performed the *in vitro* cell proliferation and *in vivo* proliferation assay in Tg(krt14:MEK2S219D-GFP) transgenic zebrafish. COS-1 cells was seeded to 24-well for 24 h and then transfected with pcDNA3-GFP or pcDNA3-MEK2S219D-GFP plasmid. The cell number of transfected COS-1 cells was counted using trypan blue assay at day 1 to day 4. The cell number of transfected with pcDNA3-MEK2S219D-GFP was found significantly higher than transfected with pcDNA3-GFP (Control) at 3 and 4 day post-transfection (Fig. 6a). The *in vivo* proliferation assay was performed using H&E and IHC staining in Tg(krt14:MEK2S219D-GFP) transgenic zbrafish. The proliferation cells were detected in papilloma (black-dots area) by H&E staining (Fig. 6b). Significantly, the much higher proportion of Ki-67 staining was detected in the epithelium of the yolk (Fig. 6c). Taking together, these data further support the contention that the constitutive activated MEK2 could induce proliferation *in vitro* and induce papilloma formation in transgenic zebrafish.

## Discussion

In this study, we established a transgenic zebrafish model to explore the role of MEK2 in skin tumor formation. Constitutive MEK1 and MEK2 phosphorylated ERK1 and induced downstream MAPK signaling activation. Transient expression of constitutive MEK2 and MEK1 in the epidermis had induced skin papillary formation at 3 dpf. Sporadic skin papilloma formation was revealed in the epidermis of Tg(krt14-MEK2S219D-GFP) transgenic zebrafish and had developed into skin tumors at 6 dpf. Treatment with U0126 could reduce skin tumor formation in Tg(krt14-MEK2S219D-GFP) transgenic zebrafish.

The functions of MEK1 and MEK2 in the Ras/Raf/Mek/Erk signaling pathway were well documented. During epidermal neoplasia formation, MEK2 and MEK1 showed mostly similar functions, but each had several differences. In zebrafish, the functions of MEK1 and MEK2 have not yet been characterized. From a phylogenic analysis, we identified and isolated zebrafish *mek1* and *mek2* cDNAs. We also isolated the downstream *erk1* cDNA to characterize the function of these two MEK proteins. An *in vitro* experiment showed that both MEK1 and MEK2 were able to phosphorylate downstream ERK1 by causing it to mutate to the constitutive active form. The ERK1 protein is then translocate from nuclei to the cytoplasm. We conclude that zebrafish MEK1 and MEK2 could activate downstream gene expression through Erk1 phosphorylation.

The role of MEK2 in skin tumor development is still being debated. MEK2 functions in mouse melanoma formation but does not contribute to skin tumor formation [17, 26, 27]. Those transgenic mice that contained chemically induced carcinomas or knockout mice were used to characterize the functions of MEK1 and MEK2. The krt14-MEK1 transgenic mice exhibited moderate hyperplasia with spontaneous skin tumor formation at 5 weeks. Downstream signaling is activated by ornithine decarboxylase (ODC) expression [7]. Surprisingly, skin tumors was sporadically formed just within 6 days in our Tg(krt14-MEK2S219D-GFP) transgenic zebrafish. The zebrafish MEK2 was sufficient to induce skin tumor formation through ERK1 activation.

In previous reports, the function of MEK1 was important in skin tumor formation. In our results, transient expression of activated MEK1 was produced papillary formation in the epidermis. The skin papilloma was also revealed in activated MEK2 expression. Although we did not establish transgenic zebrafish by expression of activated MEK1, we suggest that MEK1 also potentially to induce skin papilloma formation. Moreover, transient expression of constitutive MEK2 in the epidermis induced papillary formation. These papillae were then excised from the epidermis and placed in the medium. Collection of this skin cells and cultured in L15 medium could be continuously maintained for more than 1 week. Analysis of these skin cells showed intact nuclei, tubulin structure, and cell shape (data not shown). We suggest that these skin cells were experiencing hyper-proliferation due to MEK2 activation. This observation may be relevant to psoriasis but needs further investigation.

Ras proto-oncogenes are central regulators activating intracellular Ras-Raf-MEK-ERK signaling pathway in tumorigenesis. The zebrafish is increasingly used as a vertebrate model for studying tumorigenesis and drug screening. A transgenic approach is generally used for establishing a transgenic zebrafish. The Tg(fabp10:EGFP-kras^v12^) trangenic zebrafish specifically overexpressed high level of kras^v12^ in liver and progressed to hyperplasia and malignant tumor [22, 23]. Furthermore, the transgenic zebrafish overexpressed the BRAF^V600E^-mutated gene activated under *microphthalmia*-associated transcription factor (*mitf*) promoter induced melanocyte proliferation [25]. The transgenic zebrafish also overexpressed kras^G12V^ gene by using a pancreas specific transcription factor 1a promoter expressed in the pancreases, which caused pancreatic neoplasia [6]. The overexpression of the kras^G12V^ gene driven by cytokeratin 5 (*krt5*) or glial fibrillary acidic protein promoter induced brain tumor [13]. Mutagenesis induced by a random insertion into the genome through a Tol2 transposon-mediated transgenic approach was used as new method for studying gene function during development [14, 21, 24]. A Tol2 transposon-mediated enhancer trap contains GFP without a promoter was used for establishing transgenic lines, but no phenotype was observed in 37 transgenic lines [14]. The aforementioned method driven by the mini keratin8 promoter in the Tol2 transposon-mediated approach increased the transgenic efficiency to 16 %, but only 3.57 % of genes were inserted into the coding region [24]. The Tol2 transposon-mediated enhancer-trap using an hsp70 promoter-driven GFP was used to screen gene mutagenesis. The transgenic efficiency was increased to 70 % but only 2.73 % recessive mutations were identified [21]. To date, no gene mutation induced by Tol2 transposon-mediated enhancer trap has induced tumorigenesis. In our study, we established a Tol2 transposon-mediated Tg(Ka14:MEK2S219D-GFP) transgenic zebrafish by using a Ka14 promoter to constitutively activate MEK2S219D-GFP expression. Skip papilloma was observed at 3–6 dpf, and no other tumor type was observed in this transgenic line. We also established a Tg(Tol2:GFP) transgenic line by using Tol2 transposon-mediated approach, which did not display skin papilloma. Although we could not complicatedly exclude the possibility of a random insertion to the oncogene upstream of MEK and induction of the skin papilloma formation, we suggested that the frequency was extremely rare.

In summary, we showed that MEK2 is sufficient to induce skin papilloma formation. *In vitro* and *in vivo* experiments showed that activated MEK2 in MAPK pathway promoted downstream signaling through ERK1 phosphorylation. This activation could be inhibited by U0126 and reduced skin papilloma formation in transgenic zebrafish. This Tg(krt14-MEK2S219D-GFP) is the first established skin tumor model in zebrafish and could provide for anticancer drug screening to target the MAPK cascade.

## Conclusions

Most cancer was associated with constitutive activation of ERK signaling, which was activated ERK1/2 by MEK1 and MEK2 upon activation of receptor tyrosine kinase. Our data supported that the constitutive activation of MEK2 was sufficiently induced tumor formation in Tg(krt14:MEK2S219D-GFP) transgenic zebrafish. This transgenic zebrafish might be provided for anticancer drug screening.
